# Influence of Hydroxyapatite and Gelatin Content on Crosslinking Dynamics and HDFn Cell Viability in Alginate Bioinks for 3D Bioprinting

**DOI:** 10.3390/polym16223224

**Published:** 2024-11-20

**Authors:** Lina Maria Anaya-Sampayo, Nelly S. Roa, Constanza Martínez-Cardozo, Dabeiba Adriana García-Robayo, Luis M. Rodríguez-Lorenzo

**Affiliations:** 1 Centro de Investigaciones Odontológicas, Facultad de Odontología, Pontificia Universidad Javeriana, Bogotá 110231, Colombia; linamanaya@javeriana.edu.co (L.M.A.-S.); nelly.roa@javeriana.edu.co (N.S.R.); 2 School of Dentistry, Universidad de los Andes, Santiago 7620086, Chile; cemartinezc@uandes.cl; 3 Department of Polymeric Nanomaterials and Biomaterials, Institute Science and Technology of Polymers (ICTP-CSIC), 28006 Madrid, Spain

**Keywords:** bioinks, 3D bioprinting, alginate, gelatin, hydroxyapatite, cell viability, shear stress

## Abstract

This study investigates how varying concentrations of hydroxyapatite (OHAp) and the addition of gelatin influence the ionic crosslinking time of alginate-based bioinks, as well as the shear stress experienced by neonatal human dermal fibroblasts (HDFn) during extrusion. These factors are crucial for validating bioinks and developing viable 3D bioprinted models. Four bioink formulations were created with a 50/50 ratio of alginate to gelatin, incorporating different calcium phosphate concentrations (0%, 1%, 5%, and 10%). The bioink compositions were confirmed via Fourier Transform Infrared (FT-IR) spectroscopy, and rheological analyses evaluated their pseudoplastic behavior, printability limits, and crosslinking times. The results indicated a notable increase in the consistency index (k) from 0.32 for the 0% OHAp formulation to 0.48 for the 10% OHAp formulation, suggesting improved viscoelastic properties. The elastic modulus recovery after crosslinking rose significantly from 245 Pa to 455 Pa. HDFn experienced a shear stress of up to 1.5436 Pa at the tip during extrusion with the HDFn-ALG5-GEL5-OHAp10 bioinks, calculated at a shear rate as low as 2 s^−1^. Viability assays confirmed over 70% cell viability 24 h post-extrusion and 92% viability after 7 days for the 10% OHAp formulation, highlighting the potential of hydroxyapatite-enhanced bioinks in tissue engineering applications.

## 1. Introduction

3D bioprinting is a novel tissue engineering technology that is capable of mimicking desired tissue characteristics by combining a digital model with biomaterials and encapsulating cells to provide a three-dimensional environment, thus allowing for their homogeneous distribution within the construct [1,2]. 3D bioprinting potential is based on the capacity to yield appropriate cell distribution, key in facilitating cell interactions that should contribute to the growth of the tissue in a uniform manner [3]. Extrusion-based bioprinting is widely used in 3D bioprinting because of its affordability. Additionally, the deposition process is controlled, and a wide range of biomaterial inks can be obtained, mainly using polysaccharides [4]. Bioinks result from incorporating cells into a pseudoplastic biomaterial, which is extruded through a nozzle. The objective is to minimize the damage cells may suffer under pressure [5]. Most of the research into bioprinting is carried out by biology-based laboratories searching for quick biological responses, frequently not investigating the required bioink properties [4].

Bioprinting requires a rapid cross-linking process in the selected gel to build a tridimensional structure that may function as a scaffold. Gels made of mixtures of different components, such as the one herein presented, offer the potential of assaying different cross-linking procedures. However, ionic crosslinking with calcium ions is preferred for biomedical applications because of the mild conditions that can be used and the simplicity of ionotropic gelation, which avoids potentially toxic reagents or UV radiation. Therefore, alginate is the most widely used component in bioinks, among others, in addition to price or availability [4].

This study aimed to evaluate the printability and gelling capability of a bioink made of neonatal human dermal fibroblasts (HDFn), alginate (ALG), gelatin (GEL), and hydroxyapatite (OHAp) as a first step and proof of concept for the development of 3D bioprinted constructs intended for bone regeneration. Alginate has been selected for its ionic gelling capacity, reduced cytotoxicity, biodegradability, high availability, and low cost [6]. Alginate hydrogels can be formed by ionic crosslinking in the presence of calcium ions [7], attaining similar mechanical properties to extracellular matrices (ECM). Mimicking the ECM’s mechanical properties is one of the primary objectives intended for bone regeneration therapies [8]. However, alginate hydrogels lack biological cues that facilitate the attachment and proliferation of cells. Therefore, gelatin has been incorporated as a hydrogel component. Gelatin is a collagen derivative containing an Arg-Gly-Asp (RGD) sequence required for adhesion in multiple cell types [9]. In addition, it presents faster biodegradability than collagen. It is easy to prepare; however, it presents thermal gelation with a 35 °C phase transition that may interfere with the construct’s mechanical properties once it is placed in the cell culture incubator [10]. Hydroxyapatite has been incorporated to induce a higher mineralization capacity while increasing the mechanical stability of the construct [11,12].

Neonatal human dermal fibroblasts (HDFn) have been selected for this proof-of-concept investigation due to their significance in bioprinting. Moreover, because of their pivotal role in synthesizing and maintaining the extracellular matrix (ECM) of connective tissue [13,14] HDFs proliferate rapidly and have high ECM synthesis rates; both characteristics are valuable for cytocompatibility in vitro studies and for creating bioprinted scaffolds in tissue regeneration [13,14]. Previous studies have reported using HDFs in bioprinting with gelatin and hydroxyapatite to provide structural support and promote cellular adhesion and proliferation [15,16,17]. However, to our knowledge, the presence of calcium phosphate particles, in addition to gelatin, affecting the bioprinting limits has not been evaluated. Furthermore, the necessity of the bioink’s rapid ionic crosslinking to establish a stable construct and whether the presence of hydroxyapatite particles affects the shear stress experienced by cells on the tip during the extrusion process still needs to be defined to ultimately determine its effect on cell viability. These are essential parameters for the validation of a bioink and the creation of 3D bioprinted viable models.

## 2. Materials and Methods

### 2.1. Inks Preparation

Four different inks were prepared using the following concentrations: alginate 5% *w*/*v*, gelatin 5% *w*/*v* (ALG5-GEL5); alginate 5% *w*/*v*, gelatin 5% *w*/*v*, OHAp 1% *w*/*v* (ALG5-GEL5-OHAp1); alginate 5% *w*/*v*, gelatin 5% *w*/*v*, OHAp 5%*w*/*v* (ALG5-GEL5-OHAp5); and alginate 5% *w*/*v*, gelatin 5% *w*/*v*, OHAp 10% *w*/*v* (ALG5-GEL5-OHAp10). All inks were prepared to a final volume of 10 mL. ALG used was obtained from brown algae (Mw: 8945 g/mol, mannuronate/guluronate ratio of 0.63) (Sigma, Saint-Quentin-Fallavier, France) [7]. GEL was obtained from (Mw: 60 kDa, Sigma, Saint-Quentin-Fallavier, France). Hydroxyapatite (OHAp) had been prepared by Ca(OH)_2_ reaction with H_3_PO_4_ at a pH of 9.4. A detailed synthesis and characterization description can be found elsewhere [18]. The specific surface area was 62 m^2^/g, and the particle size D50 was 4.034 μm. Calcium chloride dihydrate (Sigma, Madrid, Spain) was obtained commercially from Sigma–Aldrich^®^ (Madrid, Spain). All materials were dissolved in DMEM low glucose (Gibco™/Invitrogen, Bleiswijk, The Netherlands). ALG was dissolved at 10% *w*/*v* at 4 °C for 24 h. Subsequently, the GEL was prepared at 10% *w*/*v* at 40 °C for 1 h; for inks with OHAp, the powder was added directly to the GEL as follows: 100 mg, 500 mg, and 1000 mg according to concentrations of 1, 5, and 10 of OHAp wt.%, respectively, and stirred for an additional 1 h. Following, the ALG solution was added and homogenized for 1 h. All inks were prepared to a final volume of 10 mL of DMEM supplemented with 10% fetal bovine serum (Gibco™/Invitrogen, Bleiswijk, The Netherlands) and an antibiotic–antimycotic mixture (Penicillin 100 units/mL, Streptomycin 100 µg/mL and 25 µg/mL of Amphotericin B, Gibco™/Invitrogen, Bleiswijk, The Netherlands). A pH measurement and adjustment (7.0) were performed before their use. Four compositions were obtained, as described in Table 1.

### 2.2. Fourier Transform Infrared Spectroscopy (FT-IR)

FT-IR analyses were performed to identify functional groups in dry samples of ALG, GEL, OHAp, and the synthesized inks. This analysis is crucial for understanding the chemical structure of the bioinks, as it allows for the identification of interactions such as the crosslinking between alginate and chloride ions [19]. The spectra were obtained utilizing an ATR-coupled Spectrum-Two Perkin Elmer spectrophotometer (PerkinElmer, Shelton, CT, USA), scanned across the 400–4000 cm^−1^ range with a resolution of 4.00 cm^−^^1^ and 16 scans [20].

### 2.3. Rheological Characterization

Rheological measurements were performed on an AR-G2 rheometer (TA Instruments, DE, USA) with a sand-blasted parallel-plate geometry (diameter: 25 mm). Samples were loaded onto the preheated at 25 °C rheometer Peltier plate using a syringe and measured after 30 s of stabilization. A shear rate sweep ranging from 0.1 to 300 s^−^^1^ was recorded. Also, the yield stress point was calculated as the intersection between viscosity and shear stress [21]. Rotational recovery was measured in each ink by subjecting it to a shear rate of 1 s^−^^1^ for 60 s, followed by a shear rate of 100 s^−^^1^ for 30 s. This process was performed twice at the maximum shear rate (100 s^−^^1^) and three times at the minimum shear rate (1 s^−^^1^) to evaluate the material’s behavior in the recovery process [21]. A Linear viscoelastic region (LVR) was determined with an oscillatory stress sweep test at 1 Hz to select a 1% strain at 25 °C and perform a frequency sweep between 0.01 and 100 Hz. After crosslinking with 1.5% CaCl_2_, a time test (conditions) at 25 °C for 10 min followed by 20 min at 37 °C was performed [21].

Shear-thinning fluids are also characterized by the Ostwald–de Waele power law model. This equation is given by $\;\tau =K{\left(\dot{\gamma }\right)}^{n}$, where *τ* refers to the shear stress, and *γ* denotes the shear rate, which is linked to the fluid’s viscosity. The parameter K defines the flow consistency, and *n* is the flow behavior index. The shear stress versus shear rate was plotted in Origin, and the allometric model was used to calculate the index [22].

### 2.4. HDFn Culture and Ink Cytotoxicity

HDFn cells (C0045C, Gibco™, Invitrogen, Bleiswijk, The Netherlands) were cultured in DMEM supplemented with 10% FBS and 1% antibiotic-antimycotic. 5 × 10^3^ cells were seeded per well in 96-well plates and allowed to attach overnight. Subsequently, ink droplets of 50 µm approximately were deposited in 1.5% *w*/*v* CaCl_2_ solution for crosslinking for 10 min and then recovered with tweezers. Inks were plated onto HDFn monolayers for 1, 3, and 7 days. This procedure only applies to the cytotoxicity assays.

Cytotoxicity of the Inks was evaluated by the alamarBlue™ test, where viable cells can reduce Resazurin, the active ingredient of alamarBlue™, to resorufin, a highly red fluorescent compound [23]. The cell culture medium was replaced every 3 days. At each time point, cell culture medium was removed, and 10 µL of alamarBlue™ (DAL1025, Gibco™, Invitrogen, Bleiswijk, The Netherlands) was added with 90 µL of cell culture medium in each well and incubated for 2 h. After incubation, scaffolds were removed from the wells, and the absorbance of the medium was measured at 570 nm. A monolayer without scaffolds served as a positive control. All experiments were performed in triplicate.

### 2.5. 3D Printing Process

The prepared inks were loaded onto a pre-heated 37 °C 3 mL cartridge (Cellink, Göteborg, Sweden). Cylindrical scaffolds of 15 mm in diameter with pores of 1.3 mm were printed using the Inkredible 3D Printer (Cellink, Göteborg, Sweden), with a 27-gauge tapered nozzle tip (0.2 mm) into 6-well cell culture plates, using a speed of 10 mm/s [6]. Once printed, each scaffold was crosslinked with 1.5% *w*/*v* calcium chloride solution (CaCl_2_, Sigma, Madrid, Spain) (pH 7.3) for 5 min and then washed with PBS. The scaffolds were kept in a cell culture medium (DMEM + FBS 10% + 1% Antibiotic/antimycotic) and incubated at 37 °C, 5% CO_2_. The medium was changed every 3 days until their use.

### 2.6. Characterization of the 3D Printed Scaffolds

#### 2.6.1. Swelling Behavior

A gravimetry test was used to evaluate the swelling behavior of the printed scaffolds [24]. The printed scaffolds were placed and submerged in cell culture medium in a 6-well plate culture and incubated at 37 °C for 24 h. Each scaffold’s initial dry weight (*w_i_*) was measured, eliminating the excess of cell culture medium with a paper towel. The swollen weight (*w_s_*) was measured at different times (1, 2, 4, 8, 19, and 24 h). The following equation was used to determine the swelling behavior:

(1) $$\%S=\frac{\left(w_{s}-w_{i}\right)}{w_{i}}\times 100$$

Equation (1). Determination of swelling behavior. Where *w_i_* is the dry weight of each scaffold, and *w_s_* is the swollen weight of each scaffold at different times.

#### 2.6.2. Degradation Behavior

A gravimetry test evaluated the printed scaffolds’ degradation behavior [25]. The initial dry weight (*w_i_*) of printed scaffolds was measured (eliminating the excess of cell culture medium with a paper towel). Then scaffolds were placed and submerged in cell culture medium in 6-well plate cultures and incubated for 1, 2, 3, 7, 14, 21, and 30 days at 37 °C. At each time point, the degradation percentage was evaluated by measuring the weight of each scaffold after incubation. The following equation was used:

(2) $$\%D=\frac{w_{i}-w_{t}}{w_{i}}\times 100$$

Equation (2). Determination of degradation percentage. *w_i_* is the printed scaffolds’ initial dry weight, and *w_t_* is the weight after the indicated incubation time.

### 2.7. 3D Bioprinting Protocol

Neonatal Human Dermal Fibroblasts (HDFn) were obtained from Gibco™ (C0045C, Gibco™, Invitrogen, Bleiswijk, The Netherlands). The cells were cultured in cell culture medium (DMEM low-glucose supplemented with 10% fetal bovine serum and 1% antibiotic-antimycotic, Gibco™, Invitrogen, Bleiswijk, The Netherlands) under 5% CO_2_ and 37 °C. When the cells reached 90% confluence, they were detached with 0.25% trypsin/EDTA (Gibco™, Invitrogen, Bleiswijk, The Netherlands). 1 × 10^6^ cells were mixed with 1 mL bioink, which was previously heated to 37 °C with a spatula. All procedures were performed under sterile conditions within a laminar flow hood (Purifier Logic+ Class II, Labconco, Kansas City, USA). A cell-laden bioink solution was added to a 3 mL cartridge for printing, and cylindrical scaffolds of 15 mm in diameter and 1.3 mm pores were obtained using the Inkredible 3D Printer (Cellink, Göteborg, Sweden), as previously described. Scaffolds were crosslinked with calcium chloride (1.5% *w*/*v*) for 10 min and then placed in 6-well plates with cell culture medium and incubated at 37 °C and 5% CO_2_ until their evaluation.

### 2.8. Calculation of Shear Stress During Bioprinting

The shear stress experienced by the cells as they passed through the cartridge and printhead tip was calculated as previously reported [6,26]. First, the flow rate (*Q*) into the cartridge or printhead tip was determined using the following equation:

(3) $$Q=\pi R^{2}V$$

where *R* is the radius (cartridge or printhead tip), and *V* is the flow velocity (defined in the 3D printer protocol). Then, the stress distribution (${\dot{\gamma }}^{n}$) due to deformation along the cartridge or printhead tip was defined with the following equation:

(4) $${\dot{\gamma }}^{n}={\left(\frac{VR^{2}}{\left(\frac{n}{3n+1}\right)\left(R\;\frac{3n+1}{n}\right)}\right)}^{n}r$$

where *R* is the maximum radius (cartridge or printhead tip), *V* is the flow velocity (defined in the 3D printer protocol), *r* is the minimum radius, and *n* is the power law index. With the flow rate (*Q*) and the stress distribution (${\dot{\gamma }}^{n}$*)* values in the cartridge or printhead tip, the shear stress (*τ*) was calculated in the cartridge or the printhead tip using the following equation as reported before [6]:

(5) $$\tau =K{\left(\dot{\gamma }\right)}^{n}$$

where *τ* is the shear stress, *K* is the flow consistency index, and *n* is the power law index.

### 2.9. Cell Viability in the Bioprinted Scaffolds

Cell viability was assessed in bioprinted scaffolds. Briefly, scaffolds were incubated for 24 h and 7 days, replacing the cell culture medium every other day. To determine viable and non-viable cells, scaffolds were treated with the Live/Dead kit (L33224, Invitrogen, Bleiswijk, The Netherlands) containing calcein AM and ethidium homodimer-1 for 30 min. Subsequently, bioprinted scaffolds were observed under an inverted fluorescent microscope (TCS SP5, Leica, Wetzlar, Alemania), where live cells stained green and dead cells red. Representative images were captured and analyzed using ImageJ2 v. 2.14.0. The cell viability was calculated with the following equation:

(6) $$\%Cell\;viability=\frac{Live\;cells\;(green)}{Dead\;cells\;\left(red\right)+Live\;cells\;(green)}\times 100$$

Equation (3). Determination of cell viability of bioprinted scaffolds.

### 2.10. Statistical Analysis

GraphPad Prism v10 software was used for the data analysis. Descriptive statistics, including mean and standard deviation, were calculated. The Shapiro–Wilk test was applied to assess the data distribution for normality. For parametric data, one-way ANOVA followed by Tukey’s post hoc test was employed, and for non-parametric data, the Kruskal–Wallis test and Dunn’s multiple comparisons test were used.

## 3. Results

### 3.1. Ink Characterization

**Figure 1:**
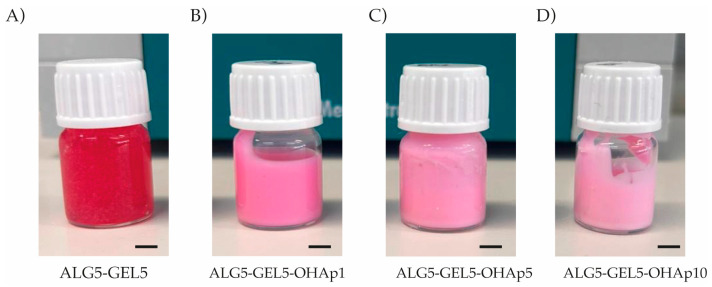
Synthetized inks with varying compositions. (**A**) 5% alginate and 5% gelatin (ALG5-GEL5); (**B**) 5% alginate, 5% gelatin, and hydroxyapatite at 1% (ALG5-GEL5-OHAp1); (**C**) 5% alginate, 5% gelatin, and hydroxyapatite at 5% (ALG5-GEL5-OHAp5); (**D**) 5% alginate, 5% gelatin, and hydroxyapatite at 10% (ALG5-GEL5-OHAp10). The abbreviations used are as follows: ALG for alginate, GEL for gelatin, and OHAp for hydroxyapatite. Each image includes a 1 cm scale bar to facilitate size reference.

Four inks with the compositions described in Table 1 were prepared. Their color and characteristics can be appreciated in Figure 1. Macroscopically, the ink composed of ALG 5% and Gel 5% was observed with a red color (Figure 1A) compared to the pink color in the inks containing hydroxyapatite (Figure 1B–D).

**Figure 2:**
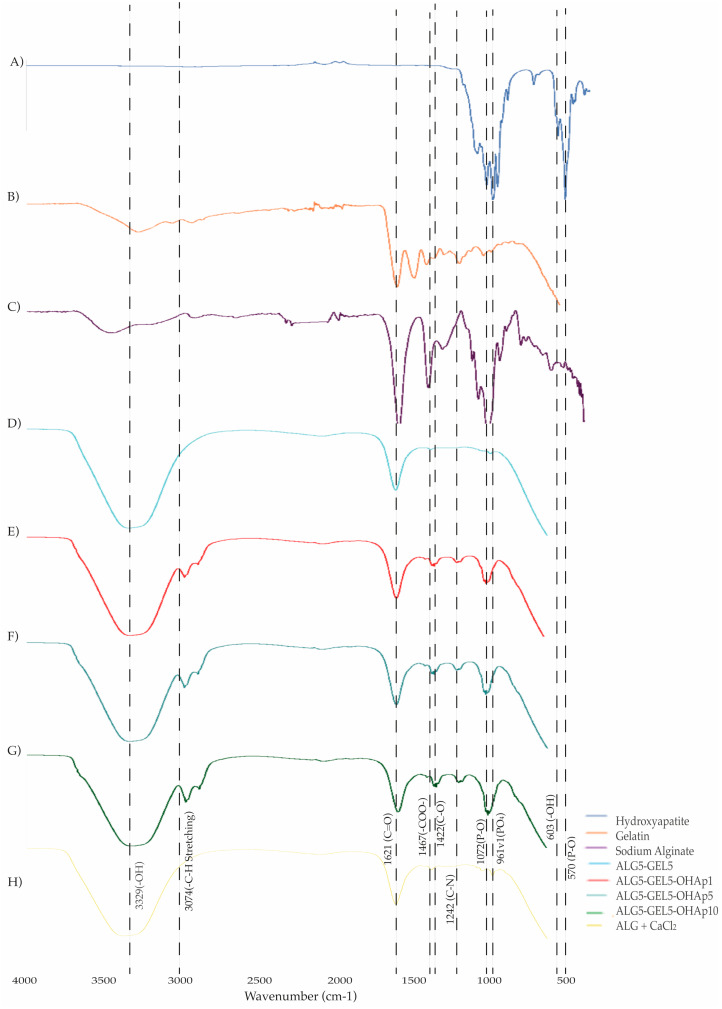
Functional groups detected in biomaterial-inks by FT-IR analysis. (**A**) Hydroxyapatite powder; (**B**) Gelatin powder; (**C**) Sodium Alginate powder; (**D**) ALG5-GEL5 (alginate 5%–gelatin 5% without hydroxyapatite); (**E**–**G**) bioinks with increasing concentrations of hydroxyapatite at 1, 5 and 10%: ALG5-GEL5-OHAp1, ALG5-GEL5-OHAp5, and ALG5-GEL5-OHAp10, respectively, and (**H**) ALG5 crosslinked with CaCl_2_. Abbreviations: ALG, alginate; GEL, gelatin; OHAp, hydroxyapatite; CaCl_2_, calcium chloride.

Fourier transform infrared spectroscopy (FT-IR) identified functional groups within the inks. In Figure 2, spectra of OHAp, GEL, ALG, and the four prepared inks are depicted with a crosslinked ink. OHAp spectrum (Figure 2A) displayed peaks at 1087, 1072, 1052, and 1032 cm^−1^ corresponding to u_3_(PO_4_). Also, peaks at 961 cm^−1^ and 874 cm^−1^ corresponded to u_1_(PO_4_), all characteristics of phosphate in an apatitic environment. GEL displayed characteristic peaks at 3329 cm^−1^ (broad) for the u (stretching vibrations) NH and OH, 1621 cm^−1^ u_C=O_, and 1242 cm^−1^ u_C-N_ from amide I, II, and III respectively; additionally, vibration stretching of the C-N bonds and the vibration of bending N-H bonds can also be observed, indicating the presence of the amide III [27]. Sodium alginate spectrum mean peaks were observed at 3390 cm^−1^ OH, 1621 cm^−1^ u _asym_ (COO), 1422 cm^−1^ u_sym_ (COO), 1050 cm^−1^ u (CC + uCO) (Figure 2C) [28]. The ALG5-GEL5 ink presented peaks at 3329 cm^−1^ and 1621 cm^−1^; hydroxyapatite functional groups were absent (Figure 2D). Following this, the inks composed of ALG5-GEL5-OHAp1, ALG5-GEL5-OHAp5, and ALG-GEL5-OHAp10 displayed the same groups as before, but additionally, with hydroxyapatite peaks, such as 1052 cm^−1^ u_3_(PO_4_) (Figure 2E–G). In summary, all the inks exhibited common groups found in gelatin and alginate. However, they varied in the presence and strength of the hydroxyapatite bands. Figure 2H displays the spectrum of a crosslinked ink and reveals the shift of the symmetric stretch vibration uCOO- associated with carboxylic acid salts from 1412–1413 cm-1 before crosslinking to 1417–1420 cm^−1^ after cross-linking. This shift can be attributed to the ionic binding [7], showing that the presence of the gelatin and hydroxyapatite does not inhibit the ionic gelation of the alginate.

### 3.2. Ink Rheological Behavior

**Figure 3:**
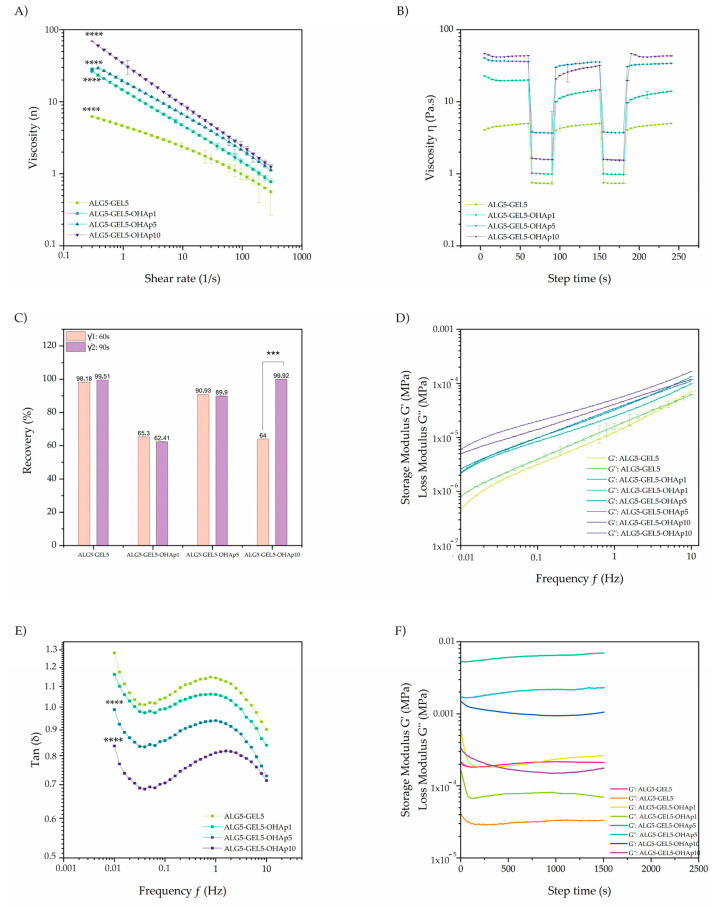
Rheological analysis of the prepared bioinks demonstrating their mechanical behavior and gelation properties. (**A**) Shear stress ramp illustrating the pseudoplastic behavior and flow properties of each bioink composition. (**B**) Shear stress recovery test showing the ability of the bioinks to recover viscosity after shear stress is removed, critical for printability. (**C**) Quantification of the recovery percentage after stress, indicating the structural resilience of each bioink. (**D**) Frequency sweep analysis displaying the storage modulus (G′) and loss modulus (G″) across a range of frequencies, providing insights into the viscoelastic properties of the bioinks. (**E**) Tan δ (loss factor) to assess the balance between elastic and viscous behavior in the bioinks. (**F**) Viscoelastic behavior (G’ and G”) after cross-linking with 1.5% CaCl_2_ for all inks. Abbreviations: ALG, alginate; GEL, gelatin; OHAp, hydroxyapatite; G′, storage modulus; G″, loss modulus. Statistical significance is indicated as *** *p* < 0.001, **** *p* < 0.0001.

All inks displayed shear thinning (pseudoplastic) behavior from a shear rate as low as 0.2 s^−1^ (Figure 3A). The OHAp incorporation into the inks significantly increased (*p* < 0.0001) its viscosity values (Figure 3A), with an over 10-fold increase for ALG5-GEL5-OHAp10 at the lower shear rates assayed, compared with ALG5-GEL5 (Figure 3A). None of the inks demonstrated a yield stress point or a dramatic decrease in the viscosity value as the shear stress increased (Figure 3A). For all inks, viscosity decreased as the shear rate increased, with an *n* index < 1 (Table 1), corresponding to a pseudoplastic behavior. Moreover, the consistency index *k* was calculated, reflecting the fluid’s initial resistance to flow [22]. The *k* index significantly increased with OHAp concentration (*p* < 0.0001 Table 1, Figure S1) compared with the OHAp-free ink.

Likewise, higher OHAp concentrations influenced the recovery percentage of the original viscoelastic properties in the inks. A recovery test was used to determine the capacity of the inks to recover from the stress induced during the printing process after 60 and 90 s. ALG5-GEL5, ALG5-GEL5OHAp1, and ALG5-GEL5OHAp5 showed a similar recovery at both evaluated times (Figure 3B,C). However, the ALG5-GEL5-OHAp10 ink evidenced a significant increase in the recovery percentage after 90 **s** compared to the recovery after 60 s (*p* < 0.0001, Figure 3B,C). OHAp also changed the solid-like behavior in the inks assessed. A frequency sweep test evaluated the behavior as liquid or solid under different deformation frequencies in all inks. A solid-like behavior is characterized by the dominance of the storage modulus (G′) over the loss modulus (G″), reflected in Tan δ (G″/G′) values < 1.

In contrast, a liquid-like behavior presents dominance of the loss modulus (G″) over the storage modulus (G′), showing Tan δ (G″/G′) values > 1. ALG5-GEL5 ink showed Tan δ values greater than 1 while the inks containing OHAp significantly decreased the Tan δ values (*p* < 0.0001, ALG5-GEL5-OHAp5, ALG5-GEL5-OHAp10) showing values lower than 1 (Figure 3D,E). The gelation process was measured at 37 °C using calcium chloride to assess the crosslinking behavior of the inks. In all tested inks, the storage modulus (G′) consistently surpassed the loss modulus (G″), particularly when crosslinked with calcium chloride. This indicates a significant enhancement in the material’s stiffness and structural integrity. Notably, inks containing OHAp demonstrated a pronounced dominance of the storage modulus. The introduction of calcium chloride increased the storage modulus, reflecting enhanced crosslinking within the material. Furthermore, inks with higher OHAp concentrations exhibited an even more significant increase in their elastic modulus, suggesting that the presence and concentration of OHAp play a crucial role in reinforcing the mechanical properties of the ink (Figure 3F) The introduction of calcium chloride increased the storage modulus, reflecting enhanced crosslinking within the material. Furthermore, inks with higher OHAp concentrations exhibited an even more significant increase in their elastic modulus, suggesting that the presence and concentration of OHAp play a crucial role in reinforcing the mechanical properties of the ink (Figure 3F).

### 3.3. 3D Printed Scaffold Swelling and Degradation Behavior

**Figure 4:**
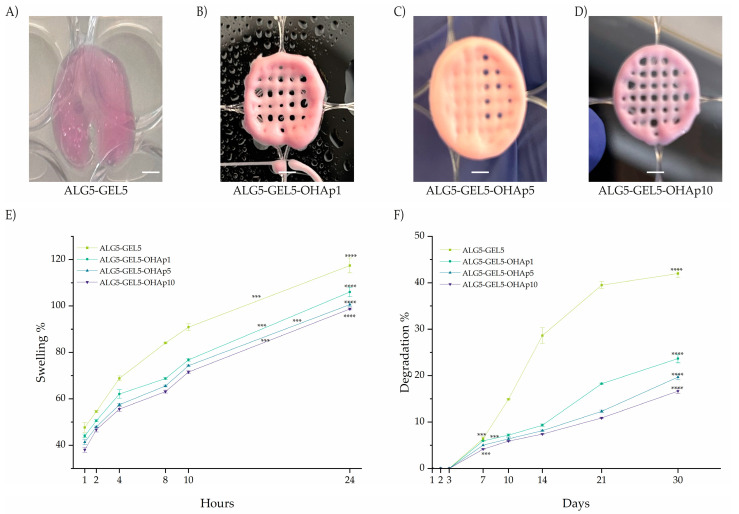
Swelling and degradation profiles of the printed scaffolds with varying hydroxyapatite (OHAp) content demonstrate the impact of OHAp on scaffold stability and water absorption capacity. (**A**–**D**) Visual representations of the different scaffold compositions: (**A**) ALG5-GEL5, (**B**) ALG5-GEL5-OHAp1, (**C**) ALG5-GEL5-OHAp5, and (**D**) ALG5-GEL5-OHAp10, highlighting structural differences and surface integrity. (**E**) Quantification of swelling percentage, showing the extent of water absorption in each scaffold composition, indicative of their hydrogel characteristics and capacity to retain moisture. (**F**) Quantification of degradation percentage over time, revealing the stability of each scaffold formulation and its suitability for long-term applications. Abbreviations: ALG, alginate; GEL, gelatin; OHAp, hydroxyapatite. Statistical significance is denoted by *** *p* < 0.001 and **** *p* < 0.0001.

The printed scaffolds’ swelling and degradation assay results are displayed in Figure 4A–D. All scaffolds showed significant swelling at 24 h (*p* < 0.0001). The highest swelling percentage was presented by the ALG5-GEL5 ink (117.41%, *p* < 0.0001), followed by ALG5-GEL5-OHAp1 (108.55%, *p* < 0.0001), ALG5-GEL5-OHAp5 (100.58%, *p* < 0.0001), and ALG5-GEL5-OHAp10 (98.70%, *p* = 0.0001). These results suggest that the increase in the inorganic phase (addition of hydroxyapatite) reduces the swelling capacity of the printed scaffolds.

Thereafter, it was observed that all the scaffolds began their degradation after 3 days of soaking (Figure 4F). The composition with the higher degradation percentage was ALG5-GEL5 (42%, *p* < 0.0001), followed by ALG5-GEL5-OHAp1 (23.67%, *p* < 0.0001), ALG5-GEL5-OHAp5 (19.67%, *p* < 0.0001), and ALG5-GEL5-OHAp10 (16.67%, *p* < 0.0001). These results also suggest that the increase in the inorganic phase reduced the degradation rate of the printed scaffolds.

### 3.4. Shear Stress During the Bioprinting Process

**Figure 5:**
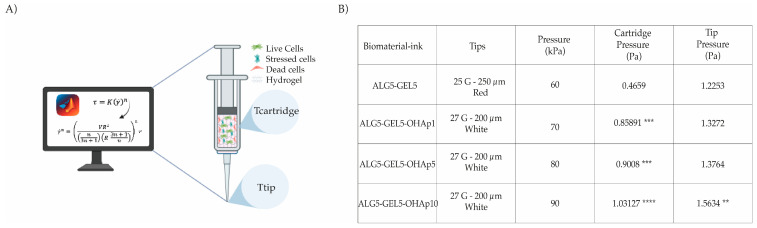
Pressure Analysis at Nozzle Tip and Syringe During Extrusion. (**A**) Simulated pressure distribution within the cartridge and at the nozzle tip, calculated using MATLAB, highlighting the mechanical forces experienced by cells during extrusion. (**B**) Comparison of extrusion pressures at the nozzle tip and syringe for different bioink formulations: ALG5-GEL5, ALG5-GEL5-OHAp1, ALG5-GEL5-OHAp5, and ALG5-GEL5-OHAp10. Statistical significance levels are marked as follows: ** *p* = 0.025, *** *p* < 0.001, **** *p* < 0.0001.

The flow rate, pressure, and shear stress experienced by the cells as they passed through the cartridge and tip of the printer can significantly affect their survival and functionality [29,30]. This study analyzed these parameters to optimize the bioprinting process for specific bioinks. Results obtained following the calculations made with Equations (5) to (7) are displayed in Figure 5.

As OHAp concentration increased, so did the extrusion pressure. This can be attributed to the material’s higher viscosity, which results from the presence of the OHAp component. Incorporating OHAp significantly elevated the extrusion pressure within the printing cartridge compared to OHAp-free (ALG5-GEL5) bioinks, for all OHAp-containing bioinks. However, there were no significant pressure changes at the tip for bioinks containing 1 and 5% of OHAP (ALG5-GEL5-OHAp1 and ALG5-GEL5-OHAp5). A slight but significant increase in pressure was observed for the bioink with the highest OHAp concentration (ALG5-GEL5-OHAp10, *p* = 0.033).

### 3.5. Ink Cytotoxicity

**Figure 6:**
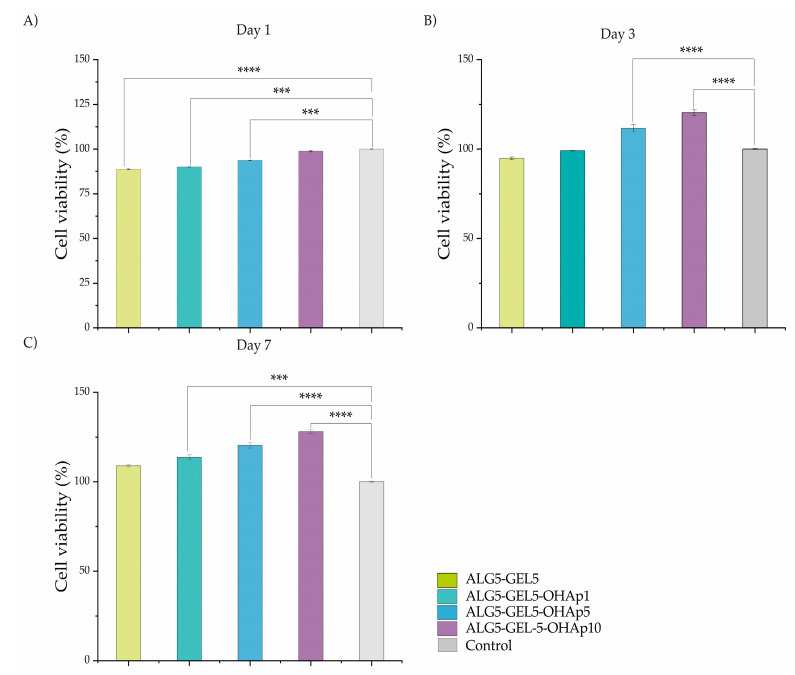
Quantitative Analysis of Cell Viability with Alamar Blue Assay. No cytotoxicity was observed for any evaluated bioink in HDFn cells across different time points. Cell viability is shown for each formulation at (**A**) 1 day, (**B**) 3 days, and (**C**) 7 days. Abbreviations: ALG, Alginate; GEL, Gelatin; HA, Hydroxyapatite. Statistical significance levels are indicated as *** *p* < 0.001 and **** *p* < 0.0001.

The cytotoxicity of the scaffold compositions was evaluated with HDFn using the alamarBlue reagent. On day 1, the cells were viable in all the evaluated compositions and revealed significantly higher viability in comparison to the control (*p* < 0.0001); however, there were significant differences (Figure 6A). On day 3 and day 7, significant differences were observed among the different compositions, demonstrating that an increase in the inorganic phase yielded more viable cells: ALG5-GEL5-OHAp10 (120.3%, and 128%, *p* < 0.0001), ALG5-GEL5-OHAp5 (111.7%, and 120%, *p* < 0.0001), and ALG5-GEL5-OHAp1 (99.08, and 113.7%, *p* = 0.0028), in comparison to the composition without hydroxyapatite ALG5-GEL5 (94.78, and 108.9, *p* = 0.002) and control (100%, *p* < 0.0001). These results show that all evaluated compositions were not cytotoxic, and increasing the amount of the inorganic phase resulted in greater viability (Figure 6B,C).

### 3.6. HDFn Viability in 3D Bioprinted Constructs

**Figure 7:**
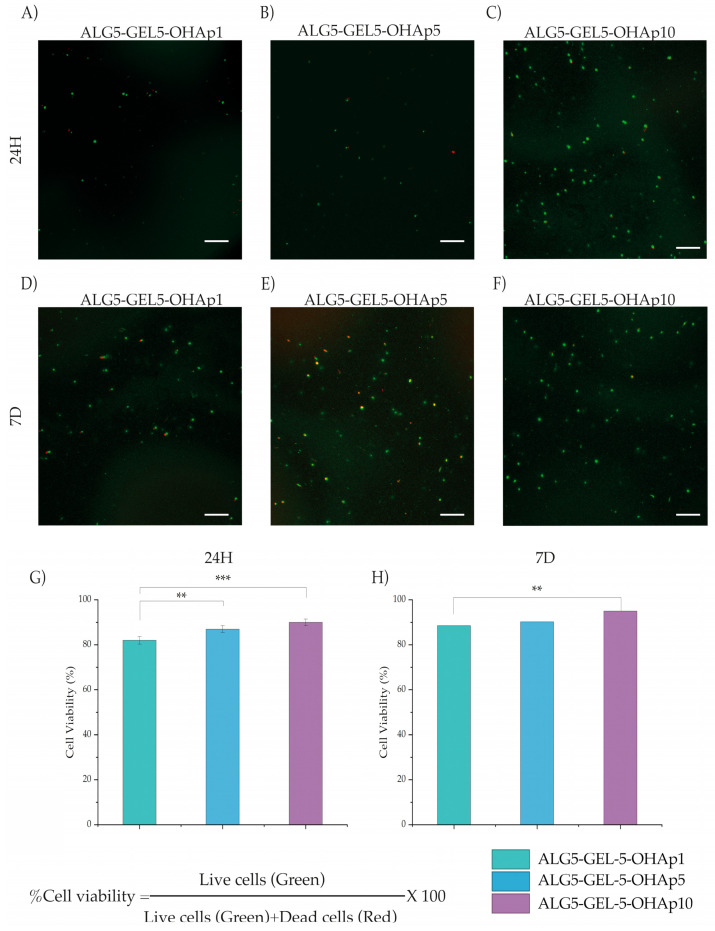
Representative Live/Dead Assay Images and Cell Viability Quantification. Images show HDFn cells in bioink formulations after (**A**,**D**) 24 h and (**B**,**E**) 7 days of bioprinting, with compositions as follows: (**A**,**D**) ALG5-GEL5-OHAp1, (**B**,**E**) ALG5-GEL5-OHAp5, and (**C**,**F**) ALG5-GEL5-OHAp10. Quantitative analysis of cell viability is displayed in (**G**) for 24 h and (**H**) for 7 days post-bioprinting. Abbreviations: ALG (Alginate), GEL (Gelatin), OHAp (Hydroxyapatite). Statistical significance is indicated by ** *p* < 0.001, *** *p* < 0.0001.

Cell viability in the bioprinted scaffolds with HDFn was evaluated using the LIVE/DEAD assay after 24 h and 7 days. Representative images showed that all printed scaffolds (ALG5-GEL5-OHAp1, ALG5-GEL5-OHAp5, and ALG5-GEL5-OHAp10) exhibited greater viability than 80% (Figure 7A–F). The scaffold with 10% OHAp showed the highest viability—90% at 24 h (*p* < 0.0001, Figure 7G) and 92% at 7 days (*p* < 0.001, Figure 7H). The presence of OHAp did not negatively affect cell viability, suggesting that the modified extrusion conditions are compatible with maintaining cell viability during bioprinting.

## 4. Discussion

Developing new bioinks, particularly those incorporating hydroxyapatite, gelatin, and alginate, represents a significant advancement in 3D bioprinting. Hydroxyapatite improves bioactivity and osteoconductivity, making it ideal for bone tissue engineering [31]. Gelatin, derived from collagen, provides a cell-friendly environment due to its biocompatibility and ability to promote cell adhesion [32]. Alginate, a naturally occurring polysaccharide, enhances the bioink’s printability by contributing viscosity and gelling properties, which are essential for maintaining structural integrity during printing, with ionic cross-linking using calcium ions being preferred for its mild conditions and simplicity [4,33].

The spectrum of the gelled ink displayed in Figure 2 revealed two peaks appearing at 1426 and 1415 cm^−^^1^ and a peak at 1025 cm^−^^1^ with a shoulder at 1071 cm^−^^1^. The first two peaks are proposed to be the asymmetric and symmetric stretch vibration of –COO associated with carboxylic acid salts [4] and are specific to ionic binding [34], thus demonstrating that the presence of gelatin and hydroxyapatite does not inhibit alginate’s ionic gelation. Furthermore, incorporating hydroxyapatite and the expected improved biological response should also offer the possibility of modifying the scaffold’s swelling capacity and modulation of degradation kinetics [29]. These findings are consistent with similar studies, such as Schütz et al., which demonstrated that alginate-based bioinks maintain structural integrity post-printing, confirming the role of ionic crosslinking in preserving scaffold shape and mechanical stability [35]. A unique finding in this research is that hydroxyapatite did not interfere with the ionic crosslinking of alginate, as confirmed by FTIR. This result aligns with studies like Benedini et al., which reported that hydroxyapatite does not hinder alginate’s gelation capacity but enhances scaffold stability and bioactivity [36].

Our results show that the presence of calcium phosphate in the scaffolds reduces the swelling compared with ceramic-free compositions. Moreover, a significant increase in calcium phosphate results in better stability (lower degradation) in the long term, which is a required parameter for further long-term experiments and applications. This observation is consistent with previous studies, such as those by Schütz et al. [35], which showed that hydroxyapatite enhances scaffold stability by decreasing swelling and degradation rates. Furthermore, Benedini et al. [36] confirmed that ceramic additives like hydroxyapatite are essential for improving scaffold performance and long-term stability, thereby supporting practical tissue regeneration applications.

However, the presence of the inorganic component has also been shown to induce a change in the viscoelastic properties of the bioink. These values should favor not only the printability but also the ink’s recovery of its viscosity. Nevertheless, all the OHAp-containing inks displayed an *n* Index between 0.47631–0.41841. In addition, according to the hydrogel behavior, the shear rate is as low as 0.2 s^−^^1^ [37]. The inclusion of hydroxyapatite in the ink composition extends the power law range. Thus, suggesting more favorable printability conditions for the hydroxyapatite-containing inks.

It has been proposed that in procedures involving the injection of living cells dispensed through syringes or catheters, the mechanical force exerted by the saline solutions used as dispersing fluid may alter the cellular membrane, compromising the cell’s viability [38]. Despite this, cell viability remains unaffected, consistent with findings in other studies that show that short-term exposure to higher shear stress does not compromise cell integrity, as observed by Malekpour et al. and Liu et al. [30,39].

This parameter is not frequently studied for syringe-injected cells in bioprinting, and it should be mandatory to report on this data for all manuscripts on bioprinting. A conical tip has been preferred for this work. According to Lucas et al.’s calculations, the tip’s shape reduces to 2 milliseconds of the stress exposure time [40]. However, it has also been described that short-term exposure to high stresses affects the proliferation potential of the cells that can survive the bioprinting process [26,41].

Reports from the literature enable us to deduce the effect stress has on cell viability and capacity for proliferation, differentiation, etc. It is cell line specific since very different values can be found for other cell lines. However, comparing literature values is often made complicated by the specific bioprinting conditions. For instance, when using 0.41 mm conical nozzles like in our studies, viability rates of 90.5–94.5% have been reported for bioprinted fibroblasts, depending on the material components of the bioink [40]. In contrast, approximately 50% viability has been achieved for chondrocyte–alginate sulfate–nanocellulose bioinks, which have a composition and viscosity closer to our bioink [42]. In our proof-of-concept model, HDFn cell viability was maintained at greater than 70% 24 h after bioprinting, indicating that the shear stress is low enough for this cell line not to compromise its viability. This is consistent with findings from Gao et al. [43], who demonstrated that human umbilical vein endothelial cells (HUVECs) maintained around 80% viability under similar extrusion-based bioprinting conditions. Both studies highlight that while some cell types, such as HDFn and HUVECs, can withstand the mechanical forces of bioprinting with minimal viability loss, the effects of shear stress remain cell-type specific.

Composites made of inorganic particles and hydrophilic polymers sometimes cannot support cell growth even though all the components are cytocompatible [44]. Cell response to scaffolds strongly depends on the material composition and the specific tested cell type. Our viability test made on the materials (Figure 6 and Figure 7) indicates a significant increase in the viability of all the compositions incorporating calcium phosphate in comparison with the ceramic-free composition. Furthermore, a considerable increase in HDFn viability was observed after 7 days of incubation for the composition containing the more significant percentage of calcium phosphate. The viability of the HDFn on the bioprinted scaffolds after 7 days of incubation within the 3D scaffold reproduces significantly greater viability for the composition with the highest concentration of calcium phosphate. Thus, in our proof-of-concept model, inorganic-loaded 3D scaffolds supported HDFn proliferation, which is critical for generating implantable, laboratory bioprinted models. Concomitantly, the bioprinting process solves a significant restriction in using 3D scaffolds, as it distributes cells within the 3D structure to generate a 3D tissue.

OHAp is involved in protein adsorption, and this process causes cell transportation into the scaffold, which is positive because it may promote cell adhesion and proliferation on scaffold surfaces [45]. Similar results on mouse fibroblast cell viability after 48 h were found for alginate–OHAp aerogels, regardless of the OHAp concentration. In general, these viability results indicate that neither the scaffold composition nor the dual processing strategy compromised cell viability. The high cell viability observed after the tests confirmed the non-toxicity of the aerogel structures and correlates with scaffolds containing the same components. Indeed, the biocompatibility of the initial components of the scaffolds (alginate and OHAp) was already reported [46]. Overall, these are promising results since cytocompatibility, and a suitable microstructure (pore size and porosity) govern the properties of an ideal scaffold [45]. The presence of OHAp in the scaffolds did not influence MSCs attachment as previously observed for structures with similar composition [47,48]. Nevertheless, for higher OHAp concentrations (16 and 24 wt%), the attachment decreases at 6 and 13 days, probably due to the high superficial roughness and irregularity that generally favor cell attachment (OHAp concentration of 8 wt%) but at much higher levels, it could make it hard for MSCs to proliferate fully attached to the aerogel. Furthermore, alginate composites combined with Ca^2+^ cations enhanced fibroblasts migration like the herein observed, probably due to changes in the regulation of genes involved in the wound healing process carried out by fibroblasts. Finally, as chemotaxis is an inherent response for cells, it was hypothesized to be involved in the cell migration process since the higher concentration of biochemical factors secreted by injured tissues activate the surrounding cells and stimulate the migration of cells. This biological response could also play a significant role in the increase of the migration of fibroblasts in the presence of the alginate-based aerogels herein observed. This study revealed that calcium ions provided by OHAp significantly enhanced cellular growth and proliferation rates [49].

One frequently mentioned limitation of bioprinting is its inability to reproduce tissue mechanics [50]. However, the purpose of using 3D bioprinted scaffolds is not to produce the mechanical properties of the tissue targeted but to simulate the extracellular matrix, providing an optimal environment that can be used by the cells to adhere, proliferate, migrate, and differentiate when MSC are used. Therefore, gelatin and calcium phosphates have been selected for this proof-of-concept work; as previously explained in the introduction, they combine the osteoconductive capacity of the hydroxyapatite with the elasticity of polysaccharides containing peptide sequences that could be recognized by cellular integrin. The results indicate that these bioinks support cell proliferation and can be used for functional tissue engineering. Human dermal fibroblasts (HDFn) were selected as a preliminary model to assess scaffold biocompatibility and structural integrity before introducing osteogenic cells [51]. As resilient and well-characterized cells, HDFn provides reliable data on cell–scaffold interactions and viability, which supports early extracellular matrix (ECM) formation and scaffold integration. Future studies will focus on osteogenic cells for targeted bone regeneration [52]. The bioinks were formulated with an optimized cell concentration (approximately 1 × 10^6^ cells/mL) to reflect physiologically relevant densities, thereby aiding scaffold maturation and potential in vivo applications. Rheological tests were conducted on the ink without adding neonatal human dermal fibroblasts (HDFn) to establish a baseline viscosity and to ensure replicability across samples. While adding HDFn cells could impact viscosity and potentially influence extrusion pressure, previous studies have shown that a cellular load of 1 × 10^6^, as selected in our experiments, has a negligible effect on the rheology of the inks, yielding statistically non-significant changes [53]. In contrast, the difference between conducting experiments with culture medium or water significantly affects these results.

Despite these promising findings, this study has limitations that should be considered. First, the research was limited to in vitro assessments, which may not accurately reflect in vivo interactions and environments. The shear stress evaluation on human dermal fibroblasts (HDFn) was restricted to specific formulations and extrusion conditions. Although this limitation affects the applicability of the results to other bioink compositions and printing parameters, it is also necessary. There are few reports on the relationship between cell-supplied and nozzle pressures, which depend on the viscoelastic properties of the bioink. Additionally, each cell line may yield different results, necessitating case-by-case studies to build the literature. While viability assays showed positive outcomes for HDFn, long-term studies on cell behavior beyond 7 days, including proliferation and matrix deposition, were not conducted.

Future research should address these limitations by incorporating in vivo models, exploring a broader range of bioink formulations that include cells during rheology testing and extended evaluation periods.

## 5. Conclusions

Incorporating calcium phosphate particles up to 10% does not inhibit the ionic crosslinking of alginate in 50/50 ALG-GEL inks. In addition, incorporating calcium phosphate particles extends the range of printability of these bioinks, induces a speedier recovery of the viscoelastic properties to the static state, and yields greater elastic modulus for the bioprinted scaffold. The pressure does not compromise the viability of HDFn experienced at the tip (up to 1.5436 Pa) during bioprinting using HDFn-ALG5-GEL5-OHAp10 bioinks, demonstrating that these bioinks support cell proliferation.

These findings highlight the clinical applicability of the developed bioinks in regenerative medicine, particularly for creating scaffolds that can facilitate tissue repair and regeneration. The scaffolds’ favorable mechanical properties and biocompatibility suggest their potential use in bone tissue engineering, where the integration of osteoconductive materials is crucial.

Looking forward, future perspectives should include comprehensive in vivo studies to assess the long-term behavior of these bioprinted scaffolds, including their biodegradability, mechanical stability, and integration with host tissues. Such studies will be essential for determining the clinical effectiveness of these scaffolds in real-world applications.

## Figures and Tables

**Table 1:** Nomenclature, composition, and power-law parameters of the prepared inks.

Ink Code	Alg (wt.%)	GEL (wt.%)	OHAp (wt.%)	K	*n*
ALG5-GEL5	5	5	0	6.918	0.56667
ALG5-GEL5-OHAp1	5	5	1	16.21055 ***	0.47134
ALG5-GEL5-OHAp5	5	5	5	22.88838 ***	0.46120
ALG5-GEL5-OHAp10	5	5	10	34.80845 ***	0.41841

*** *p* < 0.0001.

## Data Availability

Data are contained within the article and Supplementary Materials.

## Supplementary Materials

The following supporting information can be downloaded at: https://www.mdpi.com/article/10.3390/polym16223224/s1, Figure S1: Shear stress (Pa) vs. Shear rate (1/s) plot. Characterized shear-thinning behavior and determined viscosity parameters for all inks. (A) ALG5-GEL5; (B) ALG5-GEL5-OHAp1; (C) ALG5-GEL5-OHAp5; (D) ALG5-GEL5-OHAp10. ALG: Alginate, GEL: Gelatin, OHAp: Hydroxyapatite.; Figure S2: Determination of the pressure at the tip and the cartridge in bioinks. (A,B) ALG5-GEL5; (C,D) ALG5-GEL5-OHAp1; (E,F) ALG5-GEL5-OHAp5; (G,H) ALG5-GEL5-OHAp10. ALG: Alginate, GEL: Gelatin, OHAp: Hydroxyapatite.
