# Supplementary figures and images for: Influence of Hydroxyapatite and Gelatin Content on Crosslinking Dynamics and HDFn Cell Viability in Alginate Bioinks for 3D Bioprinting
Source: Polymers (Basel). 2024 Nov 20;16(22):3224. doi: 10.3390/polym16223224 (PMC11598013; doi:10.3390/polym16223224)

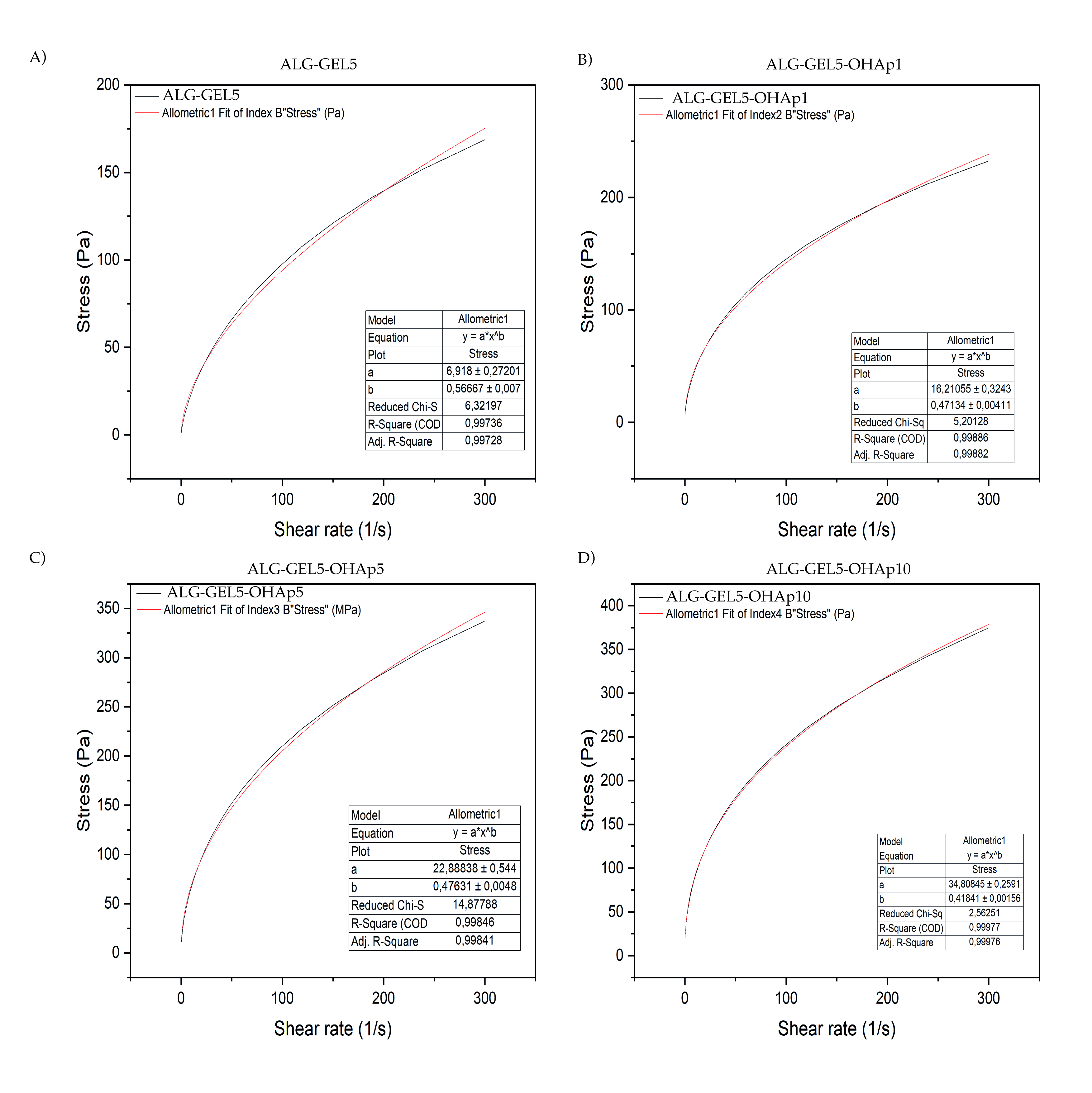

Supplement: Supplementary file 1 [file polymers-16-03224-s001.zip › polymers-3293815-supplementary/Figure S1.tif]
